# Use of rhPTH(1-84) for hypoparathyroidism during early pregnancy and lactation

**DOI:** 10.1530/EDM-22-0401

**Published:** 2023-06-19

**Authors:** E Pauline Liao, Natalie E Cusano

**Affiliations:** 1 Department of Medicine, Division of Endocrinology, Lenox Hill Hospital, New York, New York, USA

**Keywords:** Adult, Female, White, United States, Parathyroid, Bone, Novel treatment, June, 2023

## Abstract

**Summary:**

We present the first report of use of recombinant human parathyroid hormone (1-84) (rhPTH(1-84)) in a hypoparathyroid patient during early pregnancy and lactation. The patient developed postoperative hypoparathyroidism as a 28-year-old woman following total thyroidectomy for multinodular goiter. She was not well controlled with conventional therapy, and started rhPTH(1-84) in 2015 following its approval in the United States. She became pregnant in 2018 at age 40. She discontinued rhPTH(1-84) therapy at 5 weeks gestation but resumed in the postpartum period while breastfeeding. Her daughter’s serum calcium was borderline elevated at 8 days postpartum but within the normal range at 8 weeks postpartum. The patient stopped nursing at around 6 months postpartum. Her daughter is now at 4 years and 5 months of age and is healthy and meeting developmental milestones. She was again pregnant at 8 months postpartum from her first pregnancy, and she made an informed decision to continue parathyroid hormone. At 15 weeks gestation, rhPTH(1-84) was recalled in the United States due to issues with the delivery device, and she discontinued rhPTH(1-84) treatment and resumed calcium and calcitriol supplements. She gave birth to a baby boy at 39 weeks in January 2020. At 3 years and 2 months of age, he is overall healthy. Further data are needed regarding the safety of rhPTH(1-84) in pregnancy and lactation.

**Learning points:**

- rhPTH(1-84) is approved for therapy of patients with hypoparathyroidism; however, there are no data regarding the safety of treatment during nursing and pregnancy.
- There are multiple alterations in mineral metabolism during normal pregnancy and lactation.

## Background

Hypoparathyroidism is a rare endocrine disease characterized by hypocalcemia with low or undetectable parathyroid hormone (PTH) concentrations. Dr. Fuller Albright first used therapeutic parathyroid extract in 1929 (1); however, his research was abandoned until more recently (2, 3, 4, 5). Hypoparathyroidism was the only classic endocrine deficiency disease not treated with the missing hormone until approval of recombinant human parathyroid hormone (1-84) (rhPTH(1-84)) by the United States Food and Drug Administration in 2015 and the European Medicines Agency in 2017. The safety of rhPTH(1-84) in nursing and pregnancy is an area of significant interest. This is the first case report of rhPTH(1-84) use in early pregnancy and lactation in a hypoparathyroid patient.

## Case presentation

As a 28-year-old woman, the patient underwent total thyroidectomy for large multinodular goiter in 2005. Her surgery was complicated by hypoparathyroidism requiring high doses of calcium and calcitriol supplementation (3500 mg and 1 mcg daily, respectively). She had multiple hospitalizations for hypo- and hypercalcemia. In 2015, she started rhPTH(1-84), which allowed her to reduce calcium supplementation to 500 mg/day and discontinue calcitriol.

When she became pregnant in January 2018 at age 40, she discontinued rhPTH(1-84) at 5 weeks gestation, adjusted calcium to 3500 mg, and resumed calcitriol 1.0 mcg in divided doses (events and select laboratory results provided in Table 1). Her calcium supplementation remained the same; at 12 weeks, her calcitriol dose increased to 1.5 mcg and remained at this dose for the remainder of pregnancy.

**Table 1:** Timeline and selected biochemical data.

Event	Timeline	Serum calcium (mg/dL)	Calcium dose (mg/day)	Calcitriol dose (µg/day)	Other notes	
					Result	Normal range
First pregnancy	38 weeks	9.5 (8.5–10.1)	3500	1.5		
Baby girl delivered	39 weeks	10.2 (8.4–10.2)	3000	1.5	Restarted rhPTH (1-84) 75 µg daily	
Birth weight	5 lb 10 oz					
Postpartum	2 days	10.2 (8.4–10.2)	1500	1.5		
Postpartum	8 days	7.9 (8.6–10.2)	500	0	Daughter’s serum calcium: 10.6 mg/dL	8.6–10.4
Postpartum	8 weeks	9.5 (8.6–10.2)	500	0	Daughter’s serum calcium: 10.5 mg/dL	9.2–11.0
Postpartum	3 months	10.3 (8.6–10.2)	500	0	24-h urine calcium 319 mg	50–250
					rhPTH(1-84) reduced to 50 µg daily	
Postpartum	6 months	9.0 (8.6–10.2)	500	0	Nursing completed	
Postpartum	8 months	9.7 (8.6–10.2)	500	0	Patient made informed decision to continue rhPTH(1-84) 50 µg daily	
Second pregnancy	5 weeks					
Second pregnancy	12 weeks	8.5 (8.7–10.2)	500	0	Phosphorus 4.1 mg/dL	2.5-4.5
Second pregnancy	15 weeks				rhPTH(1-84) discontinued due to recall	
Second pregnancy	20 weeks	9.7 (8.7–10.2)	5000	1.5	Normal fetal anatomy scan	
Second pregnancy	27 weeks	7.6 (8.7–10.2)	5000	1.5		
Second pregnancy	28 weeks	8.5 (8.7–10.2)	6000	2.0		
Second pregnancy	32 weeks	10.3 (8.7–10.2)	6000	2.0		
Second pregnancy	34 weeks	9.3 (8.7–10.2)	6000	2.0		
Second pregnancy	36 weeks	8.9 (8.7–10.2)	4000	2.0		
Baby boy delivered	39 weeks	9.5 (8.5–10.1)	4500	1.5	APGAR scores: 8 (1 min) and 9 (5 min)	
Birth weight	5 lb 15 oz					
Postpartum	7 days	15.0 (8.5–10.1)	3500	1.0	Admission for preeclampsia	
					Phosphorus: 3.7 mg/dL	2.5–4.9
Postpartum	10 days	8.8 (8.5–10.1)	1500	0.5	Hospital discharge for preeclampsia	

She gave birth to a healthy baby girl in 2018. She developed postpartum preeclampsia with a serum calcium of 10.2 mg/dL (reference range (RR): (8.5–10.1)) at the time of hospital admission. She made the decision to resume rhPTH(1-84) while breastfeeding, at her pre-pregnancy dose of 75 mcg/day, and reduced calcium supplementation to 500 mg/day. Her daughter’s serum calcium was borderline elevated at 8 days postpartum but within the normal range at 8 weeks postpartum (Table 1). At 3 months postpartum, her rhPTH(1-84) dose was reduced to 50 mcg/day. She discontinued nursing at 6 months postpartum.

At 8 months postpartum, she found that she was again pregnant, and she decided to continue rhPTH(1-84) during this pregnancy. She made an informed decision fully aware of guidelines recommending discontinuation of rhPTH(1-84). Her dose of rhPTH(1-84) remained at 50 mcg/day with additional calcium 500 mg. At 15 weeks gestation, rhPTH(1-84) was recalled in the United States due to issues with the delivery device. She discontinued rhPTH(1-84) treatment and adjusted calcium to 5000 mg and resumed calcitriol 1.5 mcg in divided doses. Chromosomal analysis of the fetus was negative for trisomies 13, 18, and 21. Neural tube defect testing was negative. Her 20-week fetal anatomy scan was normal. Albumin-adjusted calcium levels were monitored every 2–4 weeks and ranged from 7.6 to 10.3 mg/dL (RR: 8.7–10.2). A healthy boy was born at 39 weeks 1 day, via vaginal delivery in January 2020. At 1 week postpartum, shortly after milk letdown, she developed preeclampsia and hypercalcemia (peak serum calcium 15.3 mg/dL), despite reducing calcium and calcitriol supplementation. She was readmitted for management of blood pressure and calcium and discharged after 2 days. Biochemical data for the boy neonate are lacking.

## Outcome and follow-up

Her daughter is now 4 years and 5 months of age; she is healthy and meeting developmental milestones. Her son is now 3 years and 2 months of age. He had lower extremity hypertonia in his first year, with resolution with physical therapy. He had gastroesophageal reflux for his first 18 months, with subsequent resolution. He has met his physical milestones and had some delay in spoken vocabulary, for which he was referred to (and completed) early intervention speech therapy.

## Discussion

### Alterations in mineral metabolism during pregnancy and lactation

Albumin levels decrease during pregnancy due to hemodilution, which can cause total calcium to be low, although ionized calcium remains within the normal range. Calcium metabolism and requirements are altered during pregnancy and lactation to provide adequate mineralization of the fetal skeleton. In the first trimester, calcitriol levels increase two- to five-fold in euparathyroid patients leading to increased intestinal calcium absorption (7, 8, 9). Placental contribution of 1α-hydroxylase is thought to be trivial. The rise in calcitriol is mainly due to renal 1α-hydroxylase, and pregnant women with end stage renal disease on hemodialysis have very low levels of calcitriol (10). The increase in calcitriol leads to suppression of PTH into the low-normal range in early pregnancy, with concentrations rising into the normal range by the third trimester. However, in women with low calcium intake, PTH does not suppress and increases above the normal range. Serum phosphate and magnesium remain normal during pregnancy in euparathyroid women (7, 8, 9).

PTH-related protein (PTHrP) gradually rises throughout pregnancy, increasing by three-fold by the third trimester, due to placental and breast production. During lactation, breast tissue secretes PTHrP at concentrations 1000–10 000 times higher than in patients with hypercalcemia of malignancy (11). These increased serum levels of calcitriol and PTHrP can lead to a marked reduction in calcitriol and calcium requirements during pregnancy and nursing in hypoparathyroid patients. An increase in bone resorption through estrogen withdrawal during nursing may also play a role, although the effects of estrogen withdrawal independent of nursing are difficult to study.

### Management of hypoparathyroidism during pregnancy and lactation

In pregnant women with hypoparathyroidism, calcium requirements vary; some require higher doses of calcitriol while others require a lower dose due to increased calcitriol production (8, 12, 13). It is important to monitor albumin-adjusted calcium concentrations frequently during pregnancy and adjust supplementation as needed because maternal hypo- and hypercalcemia are associated with complications including fetal morbidity and death.

Published guidelines for the management of hypoparathyroidism during pregnancy recommend monitoring serum calcium concentrations every 3–4 weeks and maintaining the albumin-corrected calcium in the lower normal range (8). Phosphorus, magnesium, and 25-hydroxyvitamin D should be maintained in the normal ranges. Discontinuation of thiazide diuretics and PTH therapy is recommended. Care of pregnant women with hypoparathyroidism should be coordinated among all care providers (endocrine, obstetrics, and pediatrics).

In the immediate postpartum period, calcium and calcitriol requirements may still be increased prior to the surge of PTHrP that occurs with the onset of lactation. Calcium levels need to be monitored and calcitriol and calcium doses should be reduced (and in some cases, discontinued) to prevent hypercalcemia. Requirements will increase when lactation wanes, although there are reports of prolonged PTHrP production long after weaning (9).

There are few published data on the effects of PTH therapy on human pregnancy or lactation. From the data presented in the rhPTH(1-84) package insert, in rats, the mean PTH concentration in milk was approximately 10 ng/mL at a treatment dose of 1000 mcg/kg/day (100 times the 100 μg/day clinical dose based on area under the curve (AUC)), 42 times lower in milk than in plasma. rhPTH(1-84) is considered pregnancy class C due to some data indicating risk in animal studies. In a pre-/post-natal study in pregnant rats given subcutaneous rhPTH(1-84) doses of 100, 300, and 1000 μg/kg/day from organogenesis through lactation (10–100 times the 100 mcg/day clinical dose based on AUC), developmental effects were observed. Entire stillborn litters were observed in the 300 μg/kg/day group (6). Whether these effects were due to hypercalcemia or PTH therapy itself was not investigated. There are two case reports of women treated with off-label PTH(1-34) during pregnancy, one with postoperative hypoparathyroidism and the second with autosomal dominant hypocalcemia type 1, with no apparent adverse effect to the mother or child (14, 15).

In summary, this is the first report of rhPTH(1-84) use in early pregnancy and lactation in a hypoparathyroid patient. One main concern during lactation is monitoring for hypercalcemia due to increased PTHrP production. Using a reduced dose of PTH may be helpful in preventing hypercalcemia and hypercalciuria. In this patient, resuming rhPTH(1-84) during nursing appears to have been without significant adverse consequence. Her daughter’s calcium levels were in the high normal range for her first 8 weeks of breastfeeding; however, she is now at 4 years and 5 months of age and is healthy and meeting developmental milestones. The patient was on rhPTH(1-84) therapy in her second pregnancy through the early part of the second trimester and gave birth to a healthy boy at 39 weeks. She developed postpartum preeclampsia with both her pregnancies, with a risk factor of advanced maternal age. The development of postpartum preeclampsia with her second pregnancy is most likely unrelated to her early pregnancy use of rhPTH(1-84). Longstanding hypercalcemia is associated with preeclampsia, although her serum calcium levels were monitored frequently and maintained within or near normal range. At 3 years and 2 months age, her son is overall healthy and has met his physical milestones with a delay in spoken vocabulary that has improved. Language delays are the most common types of developmental delay and her son’s delay is unlikely related to his very early *in utero* exposure to rhPTH(1-84), although it cannot be excluded. While this patient and her two children are overall doing well, further data are needed regarding the safety of rhPTH(1-84) in pregnancy and lactation.

## Declaration of interest

NE Cusano is a speaker and consultant for Alexion Pharmaceuticals, a consultant for Extend Biosciences, and site PI for Shire Pharmaceuticals/Takeda.

## Funding

This study did not receive any specific grant from any funding agency in the public, commercial, or not-for-profit sector.

## Patient consent

Written informed consent for publication of their clinical details was obtained from the patient.

## Author contribution statement

EPL provided direct clinical care to the patient. EPL and NEC were involved in the literature search, drafting of manuscript, and critical revisions to the final draft. All authors approved the manuscript prior to submission.
